# Comorbidity, cognitive dysfunction, physical functioning, and quality of life in older breast cancer survivors

**DOI:** 10.1007/s00520-021-06427-y

**Published:** 2021-07-21

**Authors:** Adele Crouch, Victoria L. Champion, Diane Von Ah

**Affiliations:** Indiana University School of Nursing, 600 Barnhill Drive, Indianapolis, IN 46202 USA

**Keywords:** Comorbidity, Cognitive dysfunction, Quality of life, Physical function, Breast cancer survivor, Older adults

## Abstract

**Purpose:**

Older breast cancer survivors (BCS) may be at greater risk for cognitive dysfunction and other comorbidities; both of which may be associated with physical and emotional well-being. This study will seek to understand these relationships by examining the association between objective and subjective cognitive dysfunction and physical functioning and quality of life (QoL) and moderated by comorbidities in older BCS.

**Methods:**

A secondary data analysis was conducted on data from 335 BCS (stages I–IIIA) who were ≥ 60 years of age, received chemotherapy, and were 3–8 years post-diagnosis. BCS completed a one-time questionnaire and neuropsychological tests of learning, delayed recall, attention, working memory, and verbal fluency. Descriptive statistics and separate linear regression analyses testing the relationship of each cognitive assessment on physical functioning and QoL controlling for comorbidities were conducted.

**Results:**

BCS were on average 69.79 (SD = 3.34) years old and 5.95 (SD = 1.48) years post-diagnosis. Most were stage II (67.7%) at diagnosis, White (93.4%), had at least some college education (51.6%), and reported on average 3 (SD = 1.81) comorbidities. All 6 physical functioning models were significant (*p* < .001), with more comorbidities and worse subjective attention identified as significantly related to decreased physical functioning. One model found worse subjective attention was related to poorer QoL (*p* < .001). Objective cognitive function measures were not significantly related to physical functioning or QoL.

**Conclusions:**

A greater number of comorbidities and poorer subjective attention were related to poorer outcomes and should be integrated into research seeking to determine predictors of physical functioning and QoL in breast cancer survivors.

## Introduction

Comorbidities are common among older adults; the majority (80%) have at least one comorbid condition, with the most common comorbidities being cardiovascular disease, diabetes, and arthritis [1]. In addition, individuals who have had cancer tend to report more comorbidities than those with no history of cancer [2]. Cognitive dysfunction, a common cancer-related symptom experienced by breast cancer survivors (BCS), has been associated with comorbidities in the non-oncology aging literature [3]. Findings about the relationship between comorbidities and cognitive dysfunction in older BCS specifically have begun to be addressed in the literature as well, although findings have been mixed [4–10]. Given the magnitude of comorbidities and cognitive dysfunction in older BCS, research on these associations is critical.

Additionally, both comorbidities and cognitive dysfunction have been related to decreased levels of physical functioning [11] and decreased quality of life (QoL) in older adults [12, 13]. Physical functioning is critical to independent living and QoL for older adults [14]. Decreased physical functioning can lead to the need for hospitalization, long-term care, and premature death [14]. Physical functioning and lower symptom burden is related to QoL in older adults and functional ability is identified as a critical factor in overall life satisfaction [15]. In general, poor cognitive and physical functioning as well as disruptions in QoL have far reaching implications and may impact the daily lives of older adults [13–16]. Investigation of these relationships in older BCS is reflected in the literature, although most research has been focused on older BCS in treatment and up to 2 years post-treatment, with little attention to these relationships in longer-term BCS [17].

Therefore, the purpose of this study was to examine comorbidities, objective cognitive function, and subjective cognitive function and their relationship with physical functioning and QoL in older BCS 3–8 years post-diagnosis, controlling for age and education. This study is important and may be useful for older BCS, and their families and caregivers, as well as healthcare providers, including primary care providers or geriatricians who will be caring for older BCS. Understanding the relationships between comorbidities, cognitive dysfunction, physical functioning, and QoL is essential to developing evidence-based survivorship care models that address the needs of older BCS throughout survivorship.

## Methods

This study is a secondary analysis of cross-sectional data leveraged from a large American Cancer Society-funded BCS study whose purpose was to examine QoL in younger versus older BCS (RSGPB-04–089-01, PI: Champion) [18].

### Population and data collection

The analyses for this paper focused on older BCS who were 60 years of age and older, 3–8 years post-diagnosis for stage I–IIIA breast cancer without recurrence and treated with adjuvant chemotherapy. Procedures for the original study have been published previously [18]. Briefly, eligible BCS were recruited from 97 Eastern Cooperative Oncology Group (ECOG) sites and the primary research site, a Midwestern University in the USA. Willing and eligible BCS completed mailed questionnaires documenting information including demographics, comorbidities, subjective cognitive functioning, physical functioning, and QoL. In addition, participants completed a brief telephone-based neuropsychological assessment with previously validated psychometrics [19]. Participants were paid a small incentive ($50 total) for completing the one-time questionnaires and the telephone-based neuropsychological assessment. All study procedures were reviewed and approved by the Institutional Review Boards at the primary research site and all participating ECOG sites.

### Measures

#### Demographic

Standard demographic data (i.e., age, education, race, ethnicity, marital status) were collected via an investigator-initiated self-report questionnaire.

#### Comorbidities

Comorbidities were collected via self-report survey where BCS responded yes or no to a list of 18 potential comorbid conditions including arthritis, heart disease or heart problem, high blood pressure or hypertension, stroke, serious breathing disease or problem, kidney disease or problem, high cholesterol, diabetes, leukemia or cancer (but did not include breast cancer), anxiety/panic disorders, depression, eating disorders, hip fracture, surgical replacement of joint, problem with urinary control, eye problems (other than corrective lenses), hearing problems, and other problem—please specify. For the current analyses, we used the total number of comorbidities reported.

#### Objective cognitive function

Objective cognitive function including learning, delayed recall, attention, executive function-working memory, and verbal fluency was assessed using valid and reliable neuropsychological assessments that have been used in BCS. The Rey Auditory-Verbal Learning Test (AVLT) was used to assess learning and delayed recall by completing a 15-word learning task, where the tester lists 15 words, and the participant must attempt to recall and recite [20–22]. For both learning and delayed recall, higher scores indicate better functioning [20–22]. The Digit Span Forward and Backward were used to assess attention and executive function-working memory, respectively. For the Digit Span test, the tester lists numbers in a string and the participant must recite them in order for the Forward test and must recite the numbers in reverse order for the Backward test [23, 24]. For both the Digit Span-Forward and Backward, higher scores indicate better functioning [23, 24]. The Benton Controlled Oral Word Association Test (COWA) was used to assess verbal fluency by giving the participant a letter and 1 min to produce as many words as possible that begin with that letter (excluding proper nouns) [25–27]. Potential and actual score ranges for this test vary however higher scores indicate better functioning [25–27].

#### Subjective cognitive function

The Attentional Function Index (AFI) is a 13-item scale used to assess the participants’ perceived effectiveness in activities requiring attention, working memory, and executive function, such as planning daily activities, finishing things you have started, and keeping your train of thought [28]. Potential scores can range from 0 to 130; higher scores indicate better subjective attention functioning [28]. In this study, the AFI Cronbach alpha was 0.80.

#### Physical functioning

The Physical Functioning Scale (PF-10) is a subscale of the Medical Outcomes Study 36-item Short Form Health Survey (SF-36). The PF-10 is 10 items and measures the participants’ perceived limitations of physical functioning; higher scores indicate less limitation or disability [29]. The PF-10 is an established measure of physical functioning that has shown reliability and validity in various populations including cancer patients [29]. In this study, the Cronbach alpha was 0.89.

#### Quality of life

The Index of Well-Being-Survivor (IWB) instrument measures overall QoL including life satisfaction and subjective well‐being [30]. This is a 9-item measure developed to assess specific concerns of long-term cancer survivors; higher scores indicate higher/better QoL. The IWB scale has established reliability and validity and has been widely used in cancer patients including BCS [18, 31]. In this study, the Cronbach alpha was 0.92.

### Data analysis

Descriptive statistics were calculated to describe the sample. Separate linear regression models were used to examine the relationships between the independent variables of comorbidities and both subjective cognitive function (attention) and objective cognitive assessment (learning, delayed recall, attention, executive function-working memory, and verbal fluency) and dependent variables of physical functioning (PF-10) and QoL (IWB), controlling for age and education in older BCS. A significance level of 0.05 was used for all analyses and SPSS statistical software, version 26, was used for data analysis.

An a priori power analysis was calculated based on the statistical approach described, using power estimates for a performing a linear regression model with continuous variables. Effect size estimates for estimating a model with an R2 value of 2% (small), 5% (small-medium), 13% (medium), and 26% (large) were derived from Cohen [32]. Analysis shows the sample of 335 participants has sufficient power to detect both medium and large effect sizes for any regression model ranging from one to ten independent variables, the smallest effect size (between small and medium) where all possible models will have at least 80% power. This corresponds to an effect size of *f*2 = 0.05 or *R*2 value of 5%.

## Results

Older BCS (*n* = 335) who participated in this study were 3–8 years post-diagnosis (M 5.95, SD 1.48), 63.85 years of age on average (SD 2.97), and with a mean of 13.73 (SD 2.53) years of education, indicating most BCS had at least some education beyond high school (see Table 1). On average, long-term older BCS reported having 3 (SD 1.81) comorbid conditions, with total number of comorbid conditions ranging from 0 to 12 throughout this sample. The most common comorbidities reported were hypertension (*n* = 192; 57.3%), arthritis (*n* = 186; 55.5%), and high cholesterol (*n* = 151; 45.1%). Table 1 depicts a more thorough breakdown of comorbidities reported by long-term older BCS in this study.

**Table 1:** Self-reported demographics and comorbid conditions (*n* = 335)

Age, years at breast cancer diagnosis	Mean	SD
	63.85	2.97
Education, total years	Mean	SD
	13.73	2.53
Average number of comorbid conditions	Mean	SD
	3.06	1.81
Total number of comorbid conditions	*n*	%
0	21	6.3%
1–2	107	31.9%
3–4	132	39.4%
≥ 5	75	22.4%
Comorbid condition*	*n*	%
High blood pressure or hypertension	192	57.3%
Arthritis	186	55.5%
High cholesterol	151	45.1%
Eye problems (other than corrective lenses)	83	24.8%
Depression	58	17.3%
Diabetes	55	16.4%
Heart disease or heart problem	47	14.0%
Other	45	13.4%
Surgical replacement of joint	43	12.8%
Problem with urinary control	41	12.2%
Anxiety/panic disorders	32	9.6%
Serious breathing disease or problem	27	8.1%
Hearing problems	23	6.9%
Stroke	11	3.3%
Leukemia or cancer (not breast cancer)	11	3.3%
Kidney disease or problem	14	2.4%
Eating disorders	4	1.2%
Hip fracture	3	0.9%

*SD* standard deviation

^*^(descending order from most prevalent to least prevalent condition)

The regression analyses examining number of comorbidities and objective cognitive function or subjective cognitive function scores and their relationship with physical functioning and QoL, controlling for confounders of age and education in older BCS, are displayed in Table 2. Results for each regression analyses (labeled by the independent cognitive domain variable) are described below.

**Table 2:** Regression analysis age, education, comorbidities, cognitive function measures, physical functioning, and quality of life

Objective—learning (AVLT)							
	Age	Education	Comorbidities	Learning	*F*	*R*^2^	Adjusted *r*^2^
	*Standardized β coefficient*						
Physical functioning (PF-10)	− .03	.12*	− .48**	.05	27.15**	.25	.24
QoL (IWB)	.06	.02	− .06	.04	.61	.01	− .01
Objective—delayed recall (AVLT)							
	Age	Education	Comorbidities	Delayed recall	*F*	*R*^2^	Adjusted *r*^2^
	*Standardized β coefficient*						
Physical functioning (PF-10)	− .04	.12*	− .48**	.04	26.95**	.25	.24
QoL (IWB)	.05	.03	− .06	− .04	.67	.01	.00
Objective—attention (digit span-forward)							
	Age	Education	Comorbidities	Attention	*F*	*R*^2^	Adjusted *r*^2^
	*Standardized β coefficient*						
Physical functioning (PF-10)	− .04	.13*	− .48**	.00	26.76**	.25	.24
QoL (IWB)	.06	.02	− .06	.09	1.19	.02	.00
Objective—executive function-working memory (digit span-backward)							
	Age	Education	Comorbidities	Executive function-working memory	*F*	*R*^2^	Adjusted *r*^2^
	*Standardized β coefficient*						
Physical functioning (PF-10)	− .04	.13*	− .48**	.00	26.76**	.25	.24
QoL (IWB)	.06	.01	− .06	.11*	1.52	.02	.01
Objective—verbal fluency (COWA)							
	Age	Education	Comorbidities	Verbal fluency	*F*	*R*^2^	Adjusted *r*^2^
	*Standardized β coefficient*						
Physical functioning (PF-10)	− .04	.13*	− .48**	.00	26.76**	.25	.24
QoL (IWB)	.05	.03	− .07	− .05	.70	.01	.00
Subjective—attention (AFI)							
	Age	Education	Comorbidities	Attention	*F*	*R*^2^	Adjusted *r*^2^
	*Standardized β coefficient*						
Physical functioning (PF-10)	− .04	.11*	− .42**	.23**	33.81**	.30	.29
QoL (IWB)	.05	− .03	.05	.39**	12.59**	.14	.13

^*^p < .05; **p < .01

*PF-10* physical functioning–10 sub-scale; *IWB* index of well-being; *AVLT* Rey Auditory Verbal Learning test; *COWA* Controlled Oral Word Association test; *AFI* Attention Function Index

### Physical functioning

Physical functioning scores can range from 0 to 100; in this sample, scores were on average 70.71 (SD 22.94); higher scores indicate better physical functioning or less limitation. **Learning:** The model including age, education, comorbidities, and learning was significant [*F*(4,321) = 27.15, adjusted *r*^2^ = 0.24; *p* < 0.001]. The model explained 24% of the variance of physical functioning, with education (β = 0.12, *p*˂0.05) and the number of comorbidities (β =  − 0.48, *p*˂0.001) related to physical functioning. These results indicated that more education was positively related to physical function and more comorbidities was negatively related to physical function. **Delayed recall**: Age, education, number of comorbidities, and delayed recall predicted physical functioning [*F*(4,321) = 26.95, adjusted *r*^2^ = 0.24; *p* < 0.001], explaining 24% of the variance of physical functioning. Higher levels of education (β = 0.12, *p*˂0.05) and a lower number of comorbidities (β =  − 0.48, *p*˂0.001) were related to better physical functioning. **Attention:** The model with age, education, number of comorbidities, and attention was significant [*F*(4,321) = 26.76, adjusted *r*^2^ = 0.24; *p* < 0.001], with 24% of the variance in physical functioning explained. Higher levels of education (β = 0.13, *p*˂0.05) and lower number of comorbidities (β =  − 0.48, *p*˂0.001) indicated better physical functioning. **Executive function-working memory**: The model including age, education, number of comorbidities, and executive function-working memory was significant [*F*(4,321) = 26.76, adjusted *r*^2^ = 0.24; *p* < 0.001], with 24% of the variance of physical functioning explained. Higher levels of education (β = 0.13, *p*˂0.05) and lower number of comorbidities (β =  − 0.48, *p*˂0.001) related to better physical functioning. **Verbal fluency:** Age, education, number of comorbidities, and verbal fluency predicted physical functioning [*F*(4,321) = 26.76, adjusted *r*^2^ = 0.24; *p* < 0.001], with 24% of the variance explained. Higher levels of education (β = 0.13, *p*˂0.05) and lower number of comorbidities (β =  − 0.48, *p*˂0.001) were related to better physical functioning. **Subjective attention:** The model including age, education, number of comorbidities, and subjective attention was significant [*F*(4,312) = 33.81, adjusted *r*^2^ = 0.29; *p* < 0.001], with 29% of the variance of physical functioning explained. As with previous results, higher level of education (β = 0.11, *p*˂0.05), lower number of comorbidities (β =  − 0.42, *p*˂0.001), and better subjective attention (β = 0.23, *p*˂0.001) related to better physical functioning.

### Quality of life

QoL scores ranged from 8.85 to 14.7, with older BCS in this sample reporting scores of 10.03 (SD 2.31) on average with higher scores indicating better QoL. The regression analysis models for age, education, number of comorbidities and objective cognitive measures (learning, delayed recall, attention, executive function-working memory, and verbal fluency), and QoL were not significant. However, the model for subjective attention was significant. **Subjective attention:** Age, education, number of comorbidities, and subjective attention (AFI) predicted QoL [*F*(4,310) = 12.59, *R*^2^ = 0.14, adjusted *r*^2^ = 0.13; *p* < 0.001], with 13% of the variance in QoL explained. Better subjective attention (β = 0.39, *p*˂0.001) was significantly related to better QoL.

## Discussion

Many older BCS experience multiple comorbidities as well as cognitive dysfunction following cancer diagnosis and treatment. Both comorbidities and cognitive dysfunction can have negative consequences in older BCS, including decreased physical functioning and lower QoL. This study illustrates the impact of comorbidities and cognitive dysfunction on physical functioning and QoL in older BCS.

Interestingly, the number of comorbidities reported was not related to QoL in any of the regression models, which contrasts the aging and cancer literature where comorbidities have been linked to QoL [12, 13]. However, comorbidities were significantly related to physical functioning in this sample of older BCS. The most common comorbidities for the older BCS in this study were similar to that of the general older adult population and included hypertension and arthritis. Approximately 94% (*n* = 314) of the older BCS in this study had at least one comorbid condition, as compared to 80% reported on average in the general older adult population [1]. These findings support previous literature which reports cancer survivors have more comorbidities than those without a history of cancer diagnosis and treatment [2]. Both increased comorbidities and decreased physical functioning have serious consequences in older adults [14]. These findings highlight the importance of managing comorbid conditions by healthcare providers treating older BCS. In addition, future research not only needs to examine the total number of comorbidities but should tease out the types of comorbidities that result in the greatest impairments and focus efforts on these conditions for promoting health in illness across the cancer trajectory in older BCS.

An interesting finding was that for our analyses, number of comorbidities and QoL were not related. This is in direct contrast to much of the breast cancer survivorship literature [33, 34]. However, previous literature has focused on younger or all aged BCS rather than older BCS. Researchers have shown that younger BCS often report increased symptoms and poorer quality of life than older BCS [18]. Older survivors may be more resilient than younger BCS creating less disturbance in QoL [18]. Although older BCS incur more comorbidities over time, they may be able to adapt and accept these changes as part of normal aging process more readily. However, more research is needed to fully understand the role of coping and resilience over time and its influence on QoL in longer-term survivorship for older BCS.

Subjective cognitive dysfunction, measured by the AFI, was significantly related to physical functioning. In studies among older adults, subjective cognitive dysfunction has been shown to be correlated with reports of impairment in physical functioning [35, 36]. In an older BCS study regarding trajectories of subjective cognitive decline, Mandelblatt and colleagues (2016) found that accelerated cognitive decline was associated with a decline in physical functioning in older BCS [6]. However, unlike subjective cognitive function, objective measures of cognitive function were not specifically related to physical functioning in any of the models. Similar findings have been noted in previous studies in older BCS that have examined this relationship [8–10]. Lange et al. (2014) found that in 123 older BCS with a mean age of 70 years, objective cognitive dysfunction was not related to performance status, which they hypothesized was likely due to a large proportion of BCS being in very good general health [8]. More research is needed to fully understand the link between cognitive dysfunction and physical functioning in older BCS.

Subjective cognitive dysfunction (subjective attention) was also significantly related to QoL. Similar findings have been noted in all ages of BCS [31] including those who are older [8, 37]. However, objective measures of cognitive function were not related to QoL in any of the models. Objective measures of cognitive dysfunction do not always correlate with subjective reports of QoL; Biglia and colleagues (2012) found that objective cognitive dysfunction was not related to QoL in 40 all age BCS [38]. In contrast, Lange et al. (2016) found that objective cognitive decline was associated with the QoL subscale of the Functional Assessment of Cancer Therapy, Cognitive Scale (FACT-Cog) in 119 older BCS [9]. This relationship may have been the result of overlap in examining QoL concerns specific to cognitive dysfunction with this instrument [9]. Thus, findings may vary depending on the type of objective assessment and QoL measure used. Overall, more research is needed to generate data on the relationship between cognitive dysfunction and QoL. Additionally, prospective studies are needed to fully understand the impact cognitive dysfunction has on QoL in older BCS and to begin to develop interventions to address QoL in the ever-increasing population of older BCS.

We also controlled for confounding factors that may affect our outcomes including age and education. Age was not significant among any of the models. Previous studies have shown that older age is correlated with physical function decline and increased physical limitations [39, 40]. In contrast, although older adults are more likely to have functional decline and poorer health outcomes, which can impact QoL, aging itself does not negatively influence QoL [41]. Age may have not been significant in our study because we had a predominately homogeneous age group, with BCS being 60–70 years of age. If a broader age range of older BCS had been included, there would have been more variability and a greater opportunity to examine the relationship between age, physical functioning, and QoL. Level of education was significant in the models related to physical functioning, although modestly. This could be due to education acting as a proxy or indicator of income or socioeconomic status [42]. Higher level of education, income, and/or socioeconomic status has been linked to better health outcomes, including physical functioning in the larger aging literature [43]. Future research should include a larger age range of older BCS, and potentially a closer look at the impact of education, income, and/or socioeconomic status of older BCS in relationship to physical functioning and QoL outcomes is warranted.

### Limitations

Although this study provides new information regarding comorbidity, cognitive functioning, physical functioning, and QoL in older BCS, there are limitations that should be addressed in future research. Data are cross-sectional in nature, providing a snapshot of the variables at one point in time and thus limiting the ability to determine casual relationships. A prospective, longitudinal study may have provided more insight into these relationships overtime. As a secondary data analysis, we were limited to the analysis of the measure employed in the original study. Therefore, physical function was measured by a self-report questionnaire. Future research could include objective measures of physical function for a better understanding on the impact to physical functioning in older BCS. In addition, the majority of the older BCS in this study are White (93%), which limits generalizability and the ability to address inequalities in our survivorship literature [44]. Future studies should focus on recruiting more diverse samples to ensure robust data and better understanding of racial and ethnic health-related differences [44]. Lastly, studies need to include a broader age range of older BCS to better understand the impact of age on these outcomes.

## Conclusion

Overall, older BCS with fewer years of education, more self-reported comorbidities, and worse subjective cognitive function reported worse physical functioning. Older BCS in this study who reported better subjective cognitive function reported better QoL. As evidenced by this study and others, cognitive dysfunction following cancer and treatment can have an impact on the functional ability and quality of life of patients, especially in older BCS [45]. This study provides important implications for clinical practice identifying that those older BCS with cognitive dysfunction are potentially at greater risk for decreased physical functioning and/or QoL. Additionally, findings from this study provide direction for interventions which include maintenance of physical functioning and QoL and could ultimately support independent living and even mortality.

## Data Availability

The datasets generated during and/or analyzed during the current study are available from the corresponding author on reasonable request.
